# Stromal-vascular fraction and adipose-derived stem cell therapies improve cartilage regeneration in osteoarthritis-induced rats

**DOI:** 10.1038/s41598-022-06892-3

**Published:** 2022-02-18

**Authors:** Wan-Ting Yang, Chun-Yen Ke, Kuang-Ting Yeh, Shyh-Geng Huang, Zi-Yang Lin, Wen-Tien Wu, Ru-Ping Lee

**Affiliations:** 1 Department of Orthopedics, Hualien Tzu Chi Hospital, Buddhist Tzu Chi Medical Foundation, Hualien, Taiwan; 2 Center for General Education, Cheng Shiu University, Kaohsiung, Taiwan; 3 School of Medicine, Tzu Chi University, Hualien, Taiwan; 4 Institute of Medical Sciences, Tzu Chi University, Hualien, Taiwan; 5 Master Program in Microbiology and Immunology, Tzu Chi University, Hualien, Taiwan

**Keywords:** Stem cells, Pathogenesis, Inflammation

## Abstract

This study aimed to evaluate the effects of the stromal vascular fraction (SVF) and adipose-derived stem cells (ADSCs) on cartilage injury in an osteoarthritis (OA) rat model. Sodium iodoacetate (3 mg/50 μL) was used to induce OA in the left knee joint of rats. On day 14 after OA induction, 50 μL of SVF (5 × 10^6^cells), ADSCs (1 × 10^6^ cells), or 0.9% normal saline (NS) was injected into the left knee-joint cavity of each group. The macroscopic view and histological sections revealed that the articular cartilage in the NS group was damaged, inflamed, uneven and thin, and had hyperchromatic cell infiltration. Notably, the cartilage surface had recovered to nearly normal and appeared smooth and bright on day 14 in the SVF and ADSC groups. Additionally, the white blood cell counts in the SVF and ADSC groups were higher than those in the NS group on day 14. Plasma IL-1β levels on days 7 and 14 were reduced in the SVF and ADSC groups. These results indicated that both SVF and ADSC treatments may assist in articular cartilage regeneration after cartilage injury. Cell therapy may benefit patients with OA. However, clinical trials with humans are required before the application of SVF and ADSC treatments in patients with OA.

## Introduction

Osteoarthritis (OA) is a common degenerative disorder of the articular cartilage in orthopedic elderly patients with a poor self-healing function of the knee articular cartilage, which is prone to progressive defects and dysfunction after injury^1^. In addition, the lack of effective repair function of the articular cartilage may contribute to some degenerative diseases of the knee, such as OA^1^. Signs and symptoms of OA include joint pain, stiffness, and joint swelling^2^. In OA, inflammation can occur locally or systemically, within the synovium, or with inflammatory agents circulating in the blood^1,3^. Previous studies showed that, cytokines such as interleukin (IL)-1β, IL-6, and tumor necrosis factor (TNF)-α are elevated in plasma and synovial fluid in OA patients^1,3^. The OA treatment usually involves a combination of therapies, such as medications, physical therapy, and even surgery^4,5^. However, artificial materials used in surgical treatment may have a limited validity period. Additionally, surgical procedures are complex and invasive, with high risk and varying prognosis^5^. Thus, new treatment development is warranted. Stem cells provide a rich source of cells that have a therapeutic potential in OA treatment for reducing the after-injury progression of OA^6^. Bone marrow-derived mesenchymal stem cells (BMSCs), adipose-derived stem cells (ADSCs), and synovium-derived stem cells possess the potential for cartilage formation^6^. The stromal-vascular fraction (SVF) can be extracted from fat tissue, which contains multiple cell types, including ADSCs, adipose precursor cells, fibroblasts, smooth muscle cells, and endothelial progenitor cells^7–9^. Recently, the application of BMSC, ADSC, and SVF for treating OA of the knee articular cartilage has become a hot research topic in preclinical or clinical trials and has been well established^10^. However, the therapeutic potential of the SVF for OA in comparison with that of ADSCs remains unclear. Considering in vivo treatment, the SVF and ADSCs have their own efficacy and drawbacks. SVF treatment is advantageous because the SVF has various cell types. Moreover, autologous SVF preparation may be easier than autologous ADSC preparation because, in typical preparations, autologous ADSCs are purified from the SVF^9,11,12^. Although ADSCs can be manufactured from a single cell line using modern cell manufacturing techniques, a case implant with the ADSC line has not yet been developed for use in clinical practice. ADSC treatment is advantageous because cell purity is higher in ADSCs than in the SVF. However, autologous ADSC differentiation is complicated and time-consuming. In this study, a rat model of sodium iodoacetate-induced OA was employed to compare the tissue repair effects of intra-articular injections of the SVF and ADSCs. Furthermore, chondrocytes under OA stress metabolically activate and typically increase the gene expression of several matrix components (including Col-2) to restore the extracellular matrix^13–15^. Type II collagen synthesis in OA is primarily localized in the deep zone in animals and humans^16–18^. Therefore, collagen synthesis was also analyzed by examining the pathological slides of each group.

## Results

### Sodium iodoacetate-induced OA rat model

**Figure 1:**
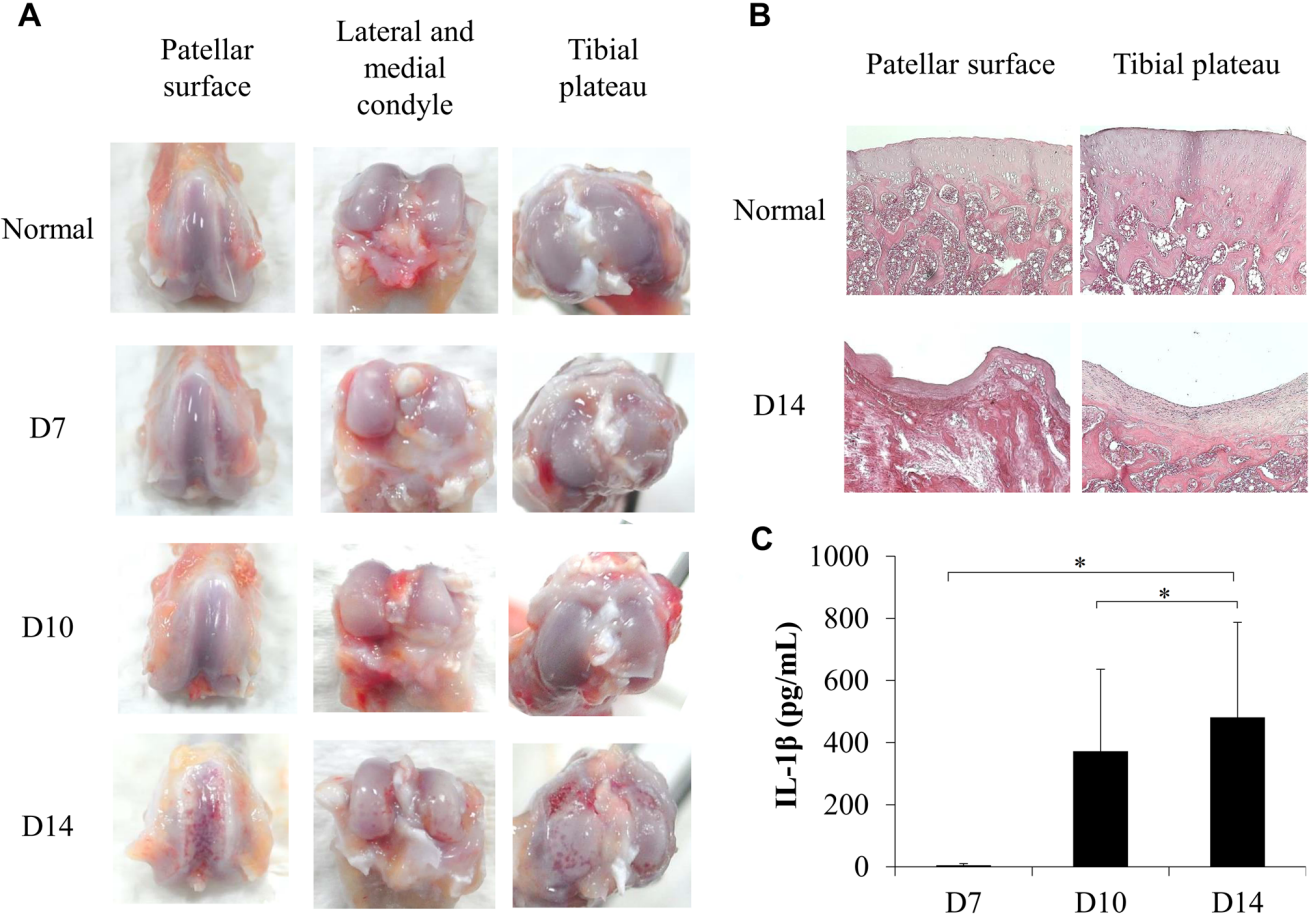
Sodium iodoacetate induced osteoarthritis in rat model. (**A**) Macroscopic view of articular cartilage of MIA-induced OA after 7, 10 and 14 days; (**B**) H&E staining of articular cartilage of MIA-induced OA after 14 days; (**C**) Plasma IL-1β level of MIA-induced OA after 7, 10 and 14 days. Data are expressed as mean ± standard error of the mean. *P < 0.05.

After the induction of OA with intra-articular injection of sodium iodoacetate, the patellar surface, lateral and medial condyles, and tibial plateau were observed on days 7, 10, and 14. Macroscopic analysis revealed that the normal articular cartilage surface was smooth and bright, whereas the sodium iodoacetate-injected cartilage was damaged, concave, and inflamed on day 14 (Fig. 1A). A thick layer of the cartilage was observed in the normal articular cartilage samples on histological analysis, whereas the sodium iodoacetate-injected cartilages were uneven, thin, and infiltrated with hyperchromatic cells on day 14 (Fig. 1B). IL-1β is a pro-inflammatory cytokine is elevated in plasma and synovial tissues in patients or animals with OA^1,19,20^. Plasma IL-1β levels increased after OA induction on day 10 (*P* = 0.03) and day 14 (*P* = 0.043) significantly by using Mann–Whitney U test (Fig. 1C). However, the histological results indicated that the OA rat model was established on day 14 after the administration of sodium iodoacetate injection.

### Macroscopic view of knee articular cartilage after treatment

**Figure 2:**
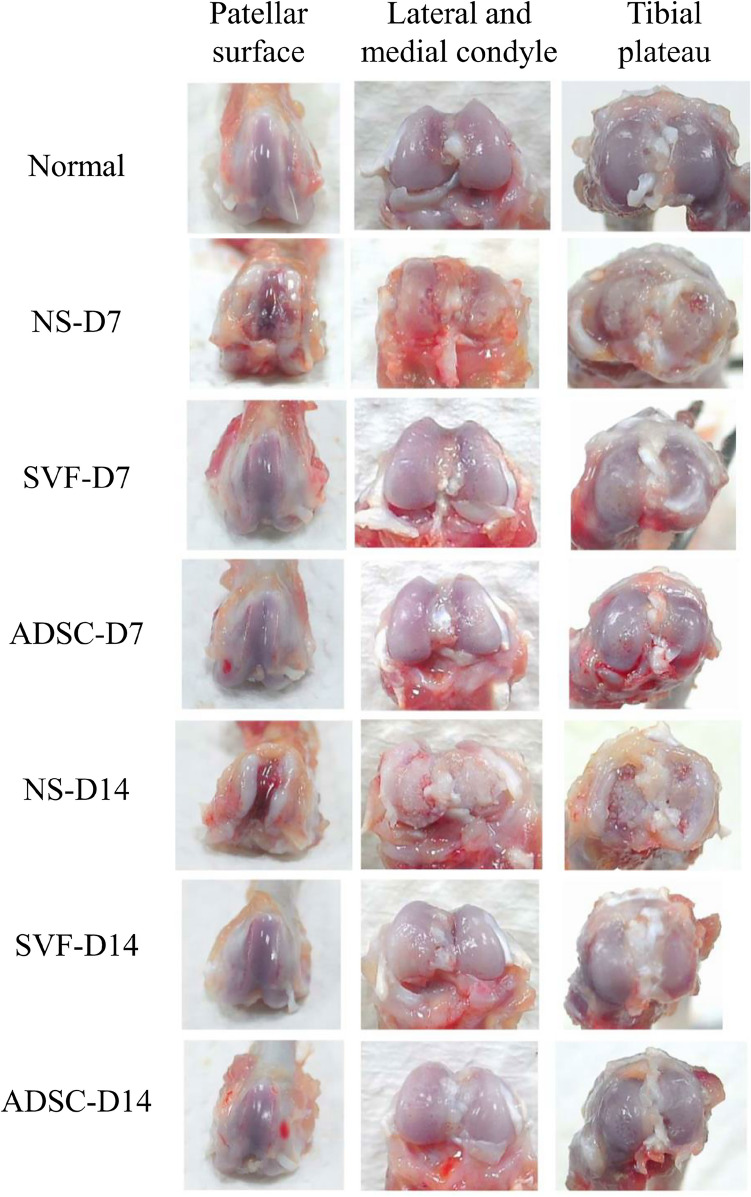
Macroscopic view of articular cartilage (patellar surface, lateral and medial condyle, tibial plateau) after NS/SVF/ADSC treatment for 7 and 14 days.

The sodium iodoacetate-induced OA rats were treated with NS, SVF, or ADSC intra-articular injection. Figure 2 indicates that on day 7 after the treatments, the cartilages of the patellar surface, lateral and medial condyles, and tibial plateau of the NS control group were severely damaged, concave, and inflamed. However, the cartilages of the patellar surface presented no signs of OA in all rats, whereas the cartilages of the condyles and the tibial plateau were slightly damaged and inflamed in the SVF and ADSC treatment groups (Fig. 2). On day 14 after the treatments, the damage and inflammation were worse than those on day 7 in the NS group, wherein the articular cartilages of the patellar surface, lateral and medial condyles, and tibial plateau were even more dilapidated. Importantly, no obvious appearance of OA was observed through the cartilages of the patellar surface, lateral and medial condyles, and tibial plateau in the SVF and ADSC treatment groups, which recovered similar to the normal articular cartilage (Fig. 2). These results suggested that SVF and ADSC treatments may contribute to cartilage repair and regeneration in OA.

### Histological observations of articular cartilage after SVF/ADSC treatment

**Figure 3:**
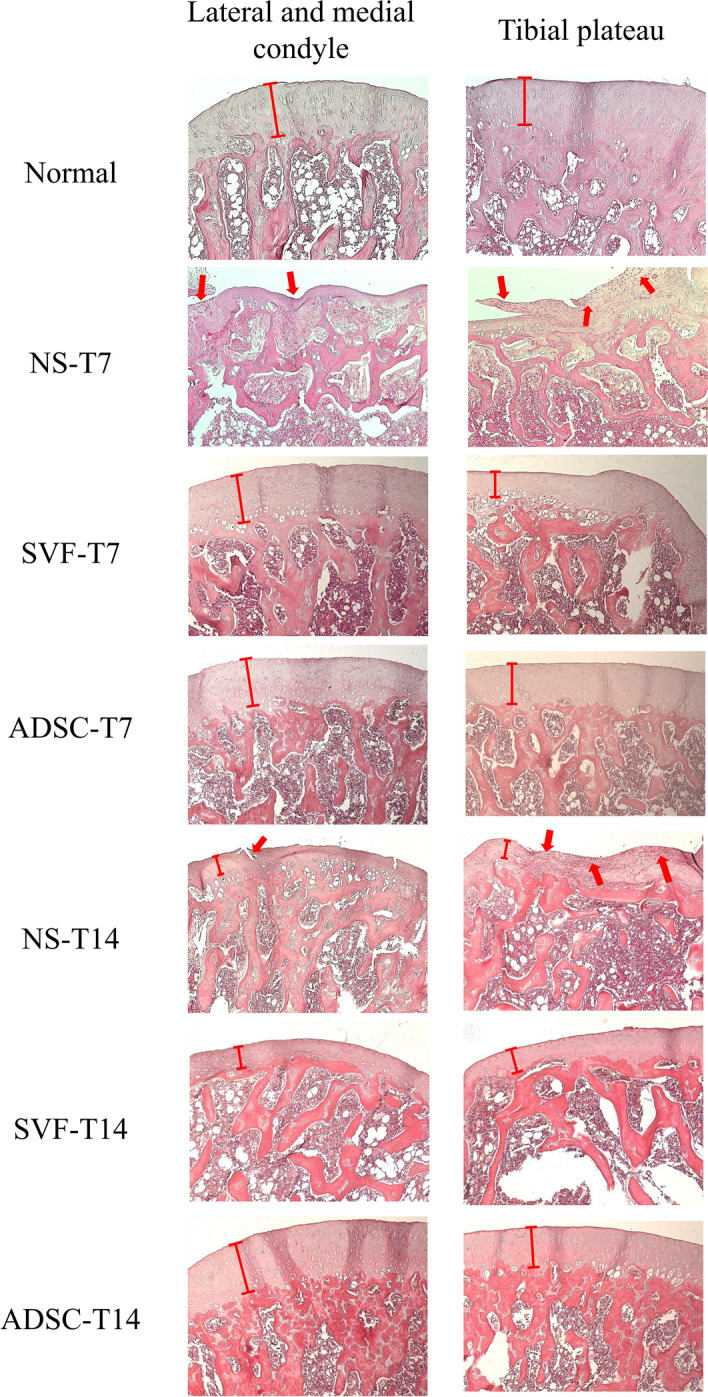
H&E staining of articular cartilage (patellar surface, lateral and medial condyle, tibial plateau) after NS/SVF/ADSC treatment for 7 and 14 days, at × 100 magnification. The hyperchromatic cells infiltration were pointed by red arrows.

H&E staining revealed that the surfaces of condyles and tibial plateau were rough, the cartilage layer was thin, and more hyperchromatic cells were observed in OA-induced tissues (Fig. 3, pointed by red arrows). On days 7 and 14 after SVF/ADSC treatment, a visible thick layer of cartilage without irregular surface was observed, which was recovered similar to that of the normal cartilage (Fig. 3, indicated with red bars). These results suggested that both SVF and ADSC treatments may contribute to cartilage regeneration in OA.

**Figure 4:**
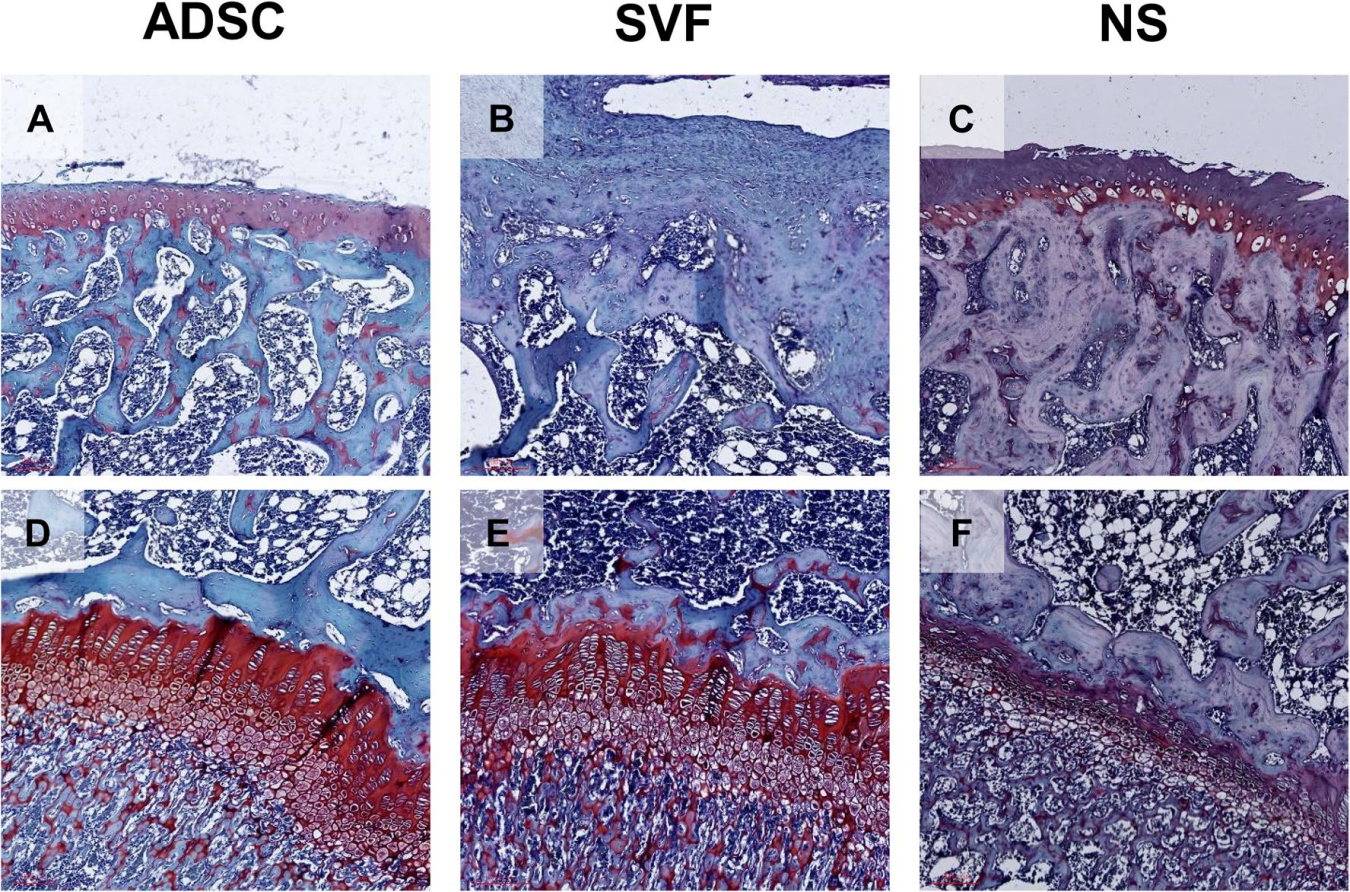
The Safranin O staining for evaluating the cartilage at × 100 magnification. (**A**–**C**) joint area. (**D**–**F**) cement line. Staining with red areas refer to articular cartilage; blue areas refer to calcified cartilage. Hyperchromatic granules within the calcified cartilage refer to bone marrow.

Safranin O staining was also used to observe the cartilages in this study. The results indicated that the amount of articular cartilage in the NS group was less than that in ADSC treatment group both at the joint and cement lines (Fig. 4A,C,D,F). The amount of articular cartilage in the SVF group was also less than that in the ADSC treatment group at the joint but approximately the same at the cement line (Fig. 4A,B,D,E). The visible differences showed that the SVF group had more calcified cartilage at the joint compared with the other two groups and also had the densest bone marrow of all the groups (Fig. 4).

### Blood sample analysis of OA rat model after treatment

**Figure 5:**
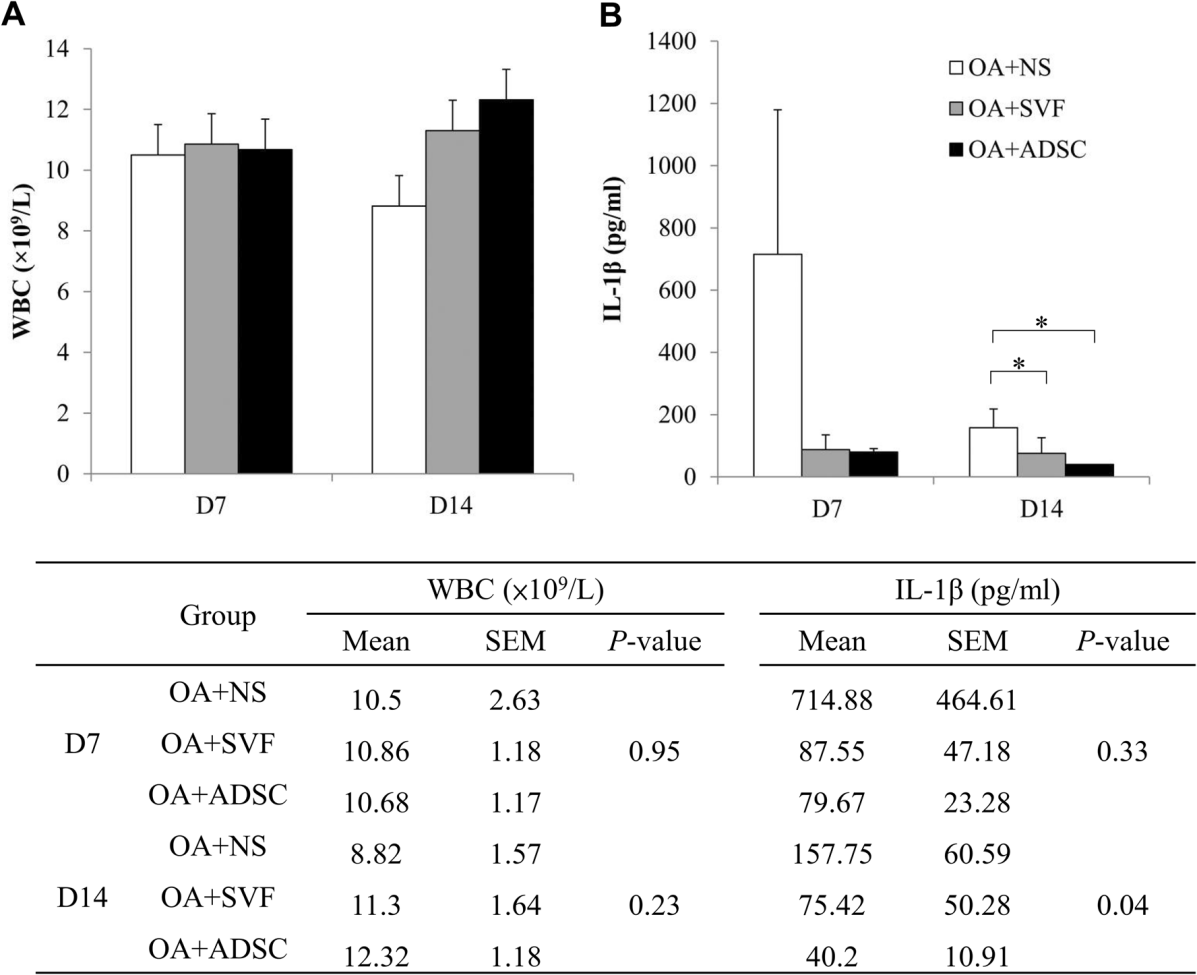
Blood analysis of (**A**) WBC count and (**B**) plasma IL-1β concentration of OA rat model after NS/SVF/ADSC treatment for 7 and 14 days. Data are expressed as mean ± standard error of the mean. Statistical analyses were performed by conducting a Mann–Whitney U test. *P < 0.05.

The white blood cell (WBC) counts were as follows: on day 14 after the NS treatment, 8.82 ± 1.57 × 10^9^/L; on day 14 after the SVF treatment, 11.3 ± 1.64 × 10^9^/L; and on day 14 after the ADSC treatment, 12.32 ± 1.18 × 10^9^/L (Fig. 5A). The average plasma IL-1β level on day 7 after the treatments (Fig. 5B) were as follows: the SVF group, 87.55 pg/mL; the ADSC group, 79.67 pg/mL; and the NS group, 714.88 pg/mL. Notably, the plasma IL-1β levels on day 14 after the SVF and ADSC treatments reduced to 75.42 pg/mL and 40.2 pg/mL, respectively (Fig. 5B) (n = 10 in each group, *P* < 0.05 by using Mann–Whitney U test). These results suggested that SVF and ADSC treatments can cause remission of the inflammatory response.

### IHC analysis

**Figure 6:**
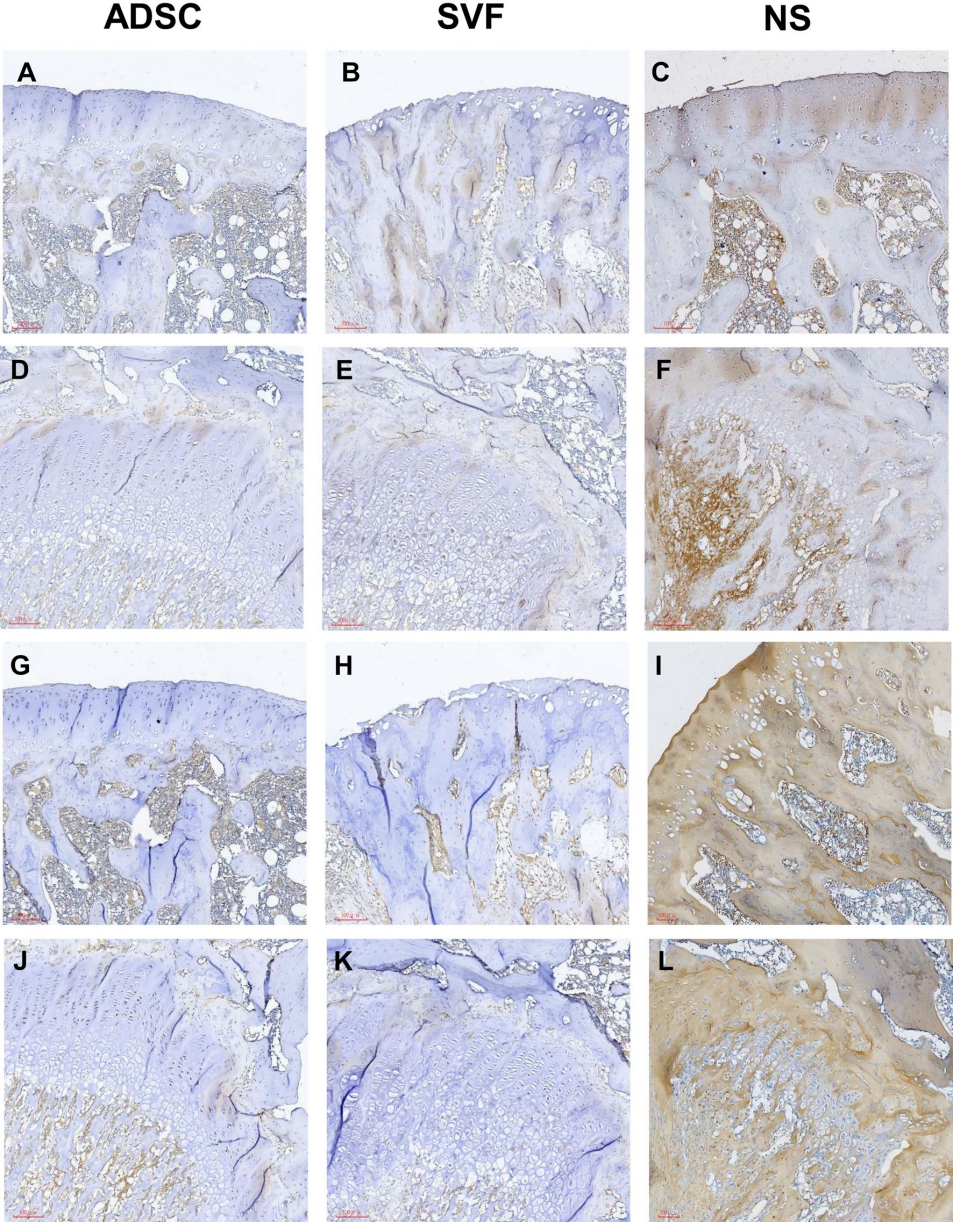
IHC staining for evaluating the COL-1 (**A**–**F**) and COL-2 (**G**–**L**) in the joint at × 100 magnification. (**A**, **B**, **C**, **G**, **H**, **I**) joint area. (**D**, **E**, **F**, **J**, **K**, **L**) cement line. Staining with brown area refer to COL-1/COL-2 positive cells.

In this study, type I collagen (COL-1) and type II collagen (COL-2) were stained using IHC. The IHC results indicated that the NS group had the highest percentage of COL-1 and COL-2 positive cells both at the joint area and cement line, and the positive cell percentages were approximately 8% and 18.72% for COL-1 and 41.8% and 36.26% for COL-2, respectively (Fig. 6C,F,I,L). The percentages of the SVF group were 8.84% and 8.49% for COL-2 positive cells, respectively, (Fig. 6H,K), which were lower than those in the ADSC group (12.76% and 10.75% for COL-2 positive cells, respectively) (Fig. 6G,J). The differences in COL-1 positive cell percentages between the SVF group and ADSC group were minuscule (within 1%; 5.53% versus 4.1% and 2.26% versus 2.98%, respectively; Fig. 6A,B,D,E).

## Discussion

In this study, the sodium iodoacetate-induced OA rat model was first well established (Fig. 1) and then was used for comparing the therapeutic effects of the SVF and ADSCs on OA. The results revealed that both the SVF and ADSCs possess therapeutic potential for cartilage regeneration. After SVF or ADSC treatment, an improvement was observed on day 7, and less tissue damage was noted during histological examination–close to full recovery–on day 14 (Figs. 2, 3). The tissue damage was more severe in the NS control group on days 7 and 14 after the treatment than in the SVF and ADSC groups (Figs. 2, 3). These results demonstrated that both the SVF and ADSCs possess similar therapeutic potential for treating OA.

After the therapeutic potential of SVF and ADSC treatments for OA were investigated, further research was conducted to explore the possible mechanisms involved in SVF and ADSC treatment. Joint pain, stiffness, swelling, and inflammation can occur locally or systemically, leading to the increase in inflammatory cytokine levels in OA patients^1–3^. In this study, WBC counts and plasma IL-1β levels were examined to determine changes in systemic inflammatory indicators after SVF and ADSC treatments. In addition, histological analysis through H&E staining was performed to determine the changes in local inflammatory responses. The H&E staining results indicated that hyperchromatic cell infiltration occurred in the NS group on day 7 after treatment and increased on day 14 (Fig. 3). The observation of hyperchromatic cell infiltration on H&E staining (Fig. 3, pointed by red arrows) indicated an inflammatory response and an increase in immune cells in the OA areas^18^. However, no such change was observed in the SVF and ADSC groups (Fig. 3).

Results of blood sample analyses revealed no difference in WBC counts between the SVF and ADSC groups on day 7 (Fig. 5A), whereas the count was higher than that in the NS group (*P* = 0.95). Whereas the WBC count decreased in the NS group on day 14, it slightly increased in the SVF and ADSC groups, without significant differences between the groups (*P* = 0.23, Fig. 5A). These results indicated that the inflammatory responses occurred in the affected area after OA induction, leading to circulatory immune cell infiltration to the affected area. Histology and macroscopic evaluations conducted after the SVF and ADSC treatments indicated that fewer immune cells infiltrated the affected area, especially the area that had almost healed on day 14. In addition, inflammatory cytokine IL-1β levels were measured to investigate the underlying process. IL-1β is a pro-inflammatory cytokine produced by joint tissues in patients or animals with OA^1,19,20^. IL-1β also activates both innate and adaptive immunity during the early stages of inflammation^21^. Studies have reported that ADSCs secrete cytokines, growth factors, and antioxidant factors, all of which regulate intracellular signaling pathways in neighboring cells^22^. After the onset of inflammation, positive regulated ADSCs cause the paracrine effect, which influences inflammatory cytokine elevation. An ADSC injection may cause the recruitment of polymorphonuclear cells, and the ADSC paracrine effect may cause these cells to upregulate cytokines such as IL-1β^22^. Therefore, we monitored the IL-1β concentration in systemic circulation to account for the anti-inflammatory response to joint tissues. Our data revealed that relative to the NS group, IL-1β levels were lower in the SVF and ADSC groups. The IL-1β levels decreased on day 7 after SVF or ADSC treatments (*P* > 0.05, Fig. 5B), which might indicate anti-inflammatory effect on the joint tissues in the early stages after treatment. These results demonstrated that the SVF or ADSC treatment reduced not only the accumulation of inflammatory cells in the OA model but also the amount of inflammatory cytokine IL-1β in the blood circulation, and had a regulating effect on immunomodulation.

In this study, we employed IHC stain to investigate the effects of ADSC and SVF during OA recovery. The data indicated that SVF had lower COL-2 expression both in the joint area and cement line (Fig. 6). Research has reported that Prolyl-4-Hydroxylase type II and type II collagen gene expressions are upregulated in OA^23^. Therefore, we proposed that OA chondrocytes reduce COL-2 expression under SVF treatment.

Figure 6 reports that type I collagen has no visual differences in the SVF and ADSC groups (Fig. 6A,B,D,E). Research has reported that the amount of type II collagen increased, and type I collagen mRNA was produced during OA progression^24^. Both the SVF and ADSCs had positive effects on OA treatment. In research investigating the effects of stem cells on OA, Van Pham et al. concluded that the SVF combined with platelet-rich plasma transplantation was an effective therapy for articular cartilage injury in a mouse model^25^. Lee et al. demonstrated that ADSCs enhanced cartilage repair and delayed the progression of OA in a rat model^26^. To our knowledge, no studies have compared the treatment effects of the SVF and ADSCs in OA animal models. This study compared the therapeutic effects between the SVF and ADSCs and revealed that the therapeutic effects on both two groups were similar in terms of the regeneration of damaged cartilage and immunomodulatory effects.

Overall, both SVF and ADSC treatments of the OA model demonstrated similar results or results with no significant difference in terms of cartilage regeneration and immunomodulating effects. The SVF comprises multiple cell types, including ADSCs, adipose precursor cells, fibroblasts, smooth muscle cells, and endothelial progenitor cells^7–9^, whereas autologous ADSCs are a pure cell population isolated from the autologous SVF^7,9,11,12^. A limitation of the present study was that although the total number of cells injected into the knee joint cavity was similar for the two groups, the number of stem cells injected into the cavity was considerably more purified for the ADSC group than for the SVF group. While the purity of stem cells administered to the ADSC group was considerably more purified than that to the SVF group, the therapeutic effects were found to be similar in both the groups. Thus, these results indicated that both the SVF and ADSCs possess nearly the same therapeutic potential for OA treatment. Notably, both the SVF and ADSCs are isolated from adipose tissues, but the cell suspension preparation methods in both groups are different. Autologous ADSC implantation is still conducted in clinical studies. Type IV hypersensitivity can be avoided by using autologous ADSC implantation instead of commercial ADSC products in the body. Autologous ADSCs are pure cell populations isolated from the autologous SVF, and differentiation is a more complicated and time-consuming process than the preparation of an autologous SVF cell suspension^9,11,12,27^. In a clinical setting, a cost- and time-efficient treatment is a more favorable option given that therapeutic effects are similar between treatments. In the present study, the same quantity of adipose tissue (initial quantity of 10 g) was used in the preparation of the SVF and ADSCs. We extracted the SVF from adipose tissue within 2 h; however, approximately 3 weeks were required to obtain the third passage of ADSCs. Nevertheless, the treatment effects of the SVF and ADSC groups were not significantly different. Thus, if no significant differences exist between the SVF and ADSCs for OA treatment, using the SVF as an autologous cell therapy is in more compliance with medical cost benefits^11,28^.

## Conclusion

This study investigated whether ADSCs and the SVF improved tissue damage in OA as well as compared the differences between these treatments. The results indicated that both SVF and ADSC therapies presented therapeutic potential for cartilage regeneration in OA. Similar therapeutic effects were observed in the SVF and ADSC groups; however, the SVF treatment was more cost- and time-efficient relative to the ADSC treatment. The aforementioned information suggests that the SVF is a favorable option for OA treatment, and it may play a particularly essential role for OA patients because most of them are socioeconomically disadvantaged older adults. Overall, this research provides a prospective suggestion for cell therapy application strategies for clinical OA patients. However, this study included no modeling of pain, which is a common symptom of OA in humans. Therefore, this study cannot provide conclusions on the effects of these treatments on pain in patients with OA. Besides, the pathohistological findings, including immunohistochemistry, were analyzed based only on visualization or semi-quantitative interpretation in this study. Furthermore, the exact percentage of individual cells and the role of these cells within the SVF should be further examined in future studies. The mechanism for reducing COL-2 expression through SVF treatment also warrants further examination. Larger animal trials, human RCTs, and phase II and III trials must be conducted before SVF and ADSC treatments can be validated and approved for patients with OA.

## Materials and methods

### Animals

A total of 30 healthy adult male Sprague–Dawley rats (age: 8–10 weeks, weight: 300–350 g) were purchased from BioLASCO Taiwan Co., Ltd (Taipei, Taiwan). The rats were housed in standard cages in a humidity- and temperature-controlled room (humidity: 55% ± 15%; temperature: 22 °C ± 1 °C) with 12-h light–dark cycles and received standard amounts of food and water. This study protocol was approved by the Institutional Animal Care and Use Committee (IACUC) of Tzu Chi University (No: 106009). All experiments were performed in accordance with relevant guidelines and regulations of IACUC.

### Isolation of SVF

Approximately 10 g of the fat tissue was obtained from the adipose tissue surrounding the rat kidneys. The adipose tissue was cut into small pieces and washed in phosphate buffered saline (PBS) and was digested using 0.2% collagenase I (Sigma-Aldrich, St. Louis, MO, USA) at 37 °C for 40 min with gentle shaking at 10-min intervals. The cell suspension was centrifuged at 700 × *g* for 10 min at 4 °C. The supernatant was discarded, and the pellet was resuspended in PBS, and filtered through a 70-μm cell strainer (Corning Inc., Corning, NY, USA). The suspension was then centrifuged at 700 × *g* for 8 min at 4 °C. The SVF pellet was resuspended in 1 mL normal saline and was ready to use. Ten microliters of the cell suspension were stained with trypan blue (Gibco) to count the total cell number by using a cell counter slide (Hausser Scientific, Horsham, PA, USA) under a microscope. The total cell count in this 1 mL SVF suspension was 1 × 10^8^ cells. And 0.05 mL from this SVF suspension were filled into a syringe; thus, each rat was intra-articularly injected with 5 × 10^6^ cells/50 μL of SVF.

### Differentiation of ADSCs

The ADSC differentiation method was modified from the previous research^29^. Approximately 10 g of fat tissue was obtained from adipose tissue surrounding the rat kidneys. The SVF pellet was isolated and resuspended in 10 mL Dulbecco’s modified Eagle’s medium/nutrient mixture F-12 (DMEM-F12; Sigma-Aldrich) containing 1% penicillin/streptomycin (Gibco, Grand Island, NY, USA) and 10% fetal bovine serum (Sigma-Aldrich). The cells extracted from 10 g of adipose tissue (the same initial quantity of fat tissue used in SVF preparation) were seeded in T75 flasks with an initial concentration of 1 × 10^6^ cells/flask and incubated at 37 °C under 5% CO_2_. The medium was changed every 3 days, and the cell growth was observed under an inverted microscope every day. When the cells achieved approximately 70%–80% confluence, the cells were detached with 0.05% trypsin–EDTA (Gibco) and passaged in T75 flasks at a concentration of 1 × 10^5^ cells/flask. Cells at the third passage were used for the experiment. The cell suspension was centrifuged at 700 × *g* for 10 min at 4 °C, and the ADSC pellet was resuspended in 1 mL NS and was ready to use. The ADSCs used for rat intra-articular injection were freshly prepared without cryopreservation. Ten microliters of the cell suspension were stained with trypan blue (Gibco) to count the total cell number by using a cell counter slide (Hausser Scientific, Horsham, PA, USA) under a microscope. Next, 0.05 mL of this cell suspension was filled into a syringe; thus, each rat was intra-articularly injected with 1 × 10^6^ cells/50 μL of ADSC.

### OA rat model and treatment

The OA rat model was induced by an intra-articular injection of sodium iodoacetate^30,31^. A total of 30 rats were randomly divided into three treatment groups: OA-NS, OA-SVF, OA-ADSC, with 10 rats in each group. A single sodium iodoacetate (Sigma-Aldrich) injection of 3 mg/50 μL was administered to the intra-articular cavity of the left knee to construct the OA rat model. All groups underwent OA induction and were treated 14 days after induction. The rats were administered intra-articular injections of 50 μL of NS (OA-NS group, as a control group), 50 μL of SVF (5 × 10^6^ cells; OA-SVF group), and 50 μL of ADSCs (1 × 10^6^ cells; OA-ADSC group) on day 14 after OA induction. Their right knees without any treatment were considered as the normal knee joints.

### Blood sample analysis

Blood samples (1 mL) were collected on days 7 and 14 after the treatments. The white blood cell (WBC) counts in the blood samples were measured using the Urit 2900 Vet Plus hematology analyzer (Diamond Diagnostics Inc., MA, USA). The blood samples were then centrifuged at 800 × *g* for 10 min at 4 °C. The plasma samples were stored at − 20 °C for later measurement of cytokine concentration. Inflammatory cytokine IL-1β levels were measured using the rat IL-1β ELISA kit (R&D Systems, Minneapolis, MN, USA) according to the manufacturer’s instructions. The optical density of each reaction was determined using the Dynex MRX II microplate reader (Chantilly, VA, USA) at 450 nm, which was converted into concentration using a standard curve. The results were expressed as pg/mL.

### Macroscopic and histological analysis of knee cartilage

Bones and cartilages of the left and right knees were obtained on days 7 and 14 after the treatments. Cartilages of the patellar surface, lateral and medial condyles, and tibial plateau were photographed using a digital camera (Nikon D70, Tokyo, Japan). The bone samples were fixed with 4% buffered formaldehyde, decalcified with a decalcifying solution, dehydrated, cleared using ethanol series to xylene, and embedded in paraffin. The paraffin blocks were cut into 5-μm tissue sections and mounted on microscopic slides for staining. The sample slides were stained with hematoxylin and eosin (H&E) for general morphology investigation and stained with safranin O for cartilage evaluation (stained with red color refer to cartilage positive). Immunohistochemistry (IHC) was performed to assess the type I collagen (COL-1) and type II collagen (COL-2) in the tissues. After dehydration, the sections on the slides were covered with coverslips and were examined microscopically. The IHC staining techniques were modified from the previous research^32^. The semi-quantification of IHC was evaluated using ImageJ software (National Institute of Health, USA). In brief, we first accessed the photo feature under the microscope section of Image J, after which we used the IHC tool box, selected the H-DAB (browner) model, adjusted the threshold, and finally measured the percentage of spots that appeared on the software interface during the progression. Semi-quantitative data are expressed as a percentage of positive cells in IHC.

### Statistical analysis

Statistical analyses were performed by conducting normality tests and nonparametric statistical tests. Kolmogorov–Smirnov and Shapiro–Wilk tests were performed to test for normality. All data exhibited a normal distribution. A comparison of the groups’ blood WBC counts and IL-1β levels was performed by conducting a Kruskal–Wallis test with IBM SPSS software version 22 (SPSS, IBM, Chicago, IL, USA). The Mann–Whitney U test was used to compare differences between two independent groups. The data were presented as mean ± standard error of the mean. *P* values less than 0.05 were considered statistically significant.

### Ethics declarations

Ethical approval for this study was approved by the Institutional Animal Care and Use Committee (IACUC) of Tzu Chi University (No: 106009). All experiments were performed in accordance with relevant guidelines and regulations of IACUC.

## Data Availability

All data generated or analyzed data during this study are included in this published article. This study followed the recommendations in the ARRIVE guidelines.
